# If Practice Makes Perfect, How Do We Perfect Our Practice?

**DOI:** 10.1016/j.jscai.2022.100306

**Published:** 2022-04-19

**Authors:** Kathleen E. Kearney

**Affiliations:** Department of Medicine, University of Washington Medical Center, Seattle, Washington

**Keywords:** Certification, education, interventional cardiology, vascular access

To paraphrase a mentor, patients leaving the catheterization lab care about 2 things—the food and their bruise—and we can only control one of those. Vascular access remains a common source of complications ranging from annoying to deadly, particularly when dealing with large-bore access. While few technical aspects of interventional cardiology have broad consensus, there is a growing amount of data and training on best practices for percutaneous vascular access. This focus on standardizing access techniques^1^ feels natural, as few aspects of practice have such early and direct feedback and are so clearly linked with our performance on the backdrop of patient-related factors.

The axillary artery is a newer addition to the armamentarium of the interventional cardiologist. Long held as a noncompressible site only by our surgical colleagues via cut-down and direct puncture, safe and effective percutaneous axillary access has now been demonstrated.^2^ While there has been considerable growth in interest and experience in percutaneous axillary access, it is not yet standard in training programs, and many operators may be crafting this skill after graduating fellowship. Additionally, technical gains and modern practice often outpace those of large-scale data in technical fields, so defining competency in a new technique proves challenging. The SCAI Position Statement on Best Practices for Percutaneous Axillary Arterial Access and Training published in this issue of *JSCAI*^3^ serves both as a clinical overview and suggests a pathway to learning this skill.

## Step 1: Defining perfection

The authors of this SCAI statement outline a pathway for developing percutaneous axillary arterial access skills safely and effectivity. A more difficult question may be, who is responsible for declaring competency in a new technique? When coronary balloon angioplasty was introduced to new operators via a short course a few decades ago, we typically had an operating room on standby for acute vessel closure—while not so far away in time, we can all agree that we have come a long way in defining our training requirements since then. The industry has some responsibility and incentives for training interventionalists on device-specific issues, but the question of who polices our techniques remains a tricky one. Societies such as SCAI have the important role of bringing together experience and developing guidelines, but it is less clear how these statements should be implemented and how unacceptable deviations in practice are defined or managed. Notwithstanding this gray zone surrounding most of our techniques, written guidance for the novice operator is an important place to start. We may expect patients to vote with their feet, but in reality, even the most medically literate patient cannot know all the pertinent questions to ask, and few are seeking a specific skill. In the end, perhaps it falls on each of us as interventional operators to pursue iterative self-improvement and to raise the standard of care in each of our own catheterization labs.

## A pathway to excellence

How do operators build off expert experience to safely offer percutaneous axillary access? These guidelines serve as a great summary of known issues and areas of uncertainty. Such documents are also useful to serve as a checklist before entering a case that we don’t practice daily. I expect this SCAI position statement will be widely referenced and integral to our fellows’ reading lists and is a good first step in incorporating percutaneous axillary access in your program.

Next, most of us learn best from observing an expert in action. While live cases occasionally offer this opportunity, it seems recorded content may be underutilized for step-by-step instruction of best practices. As internal medicine residents, many of us reviewed videos on thoracenteses in the middle of the night as a quick review; if nothing else, allowing easy access to a practical demonstration that was then performed supervised and ultimately unsupervised at the bedside and providing a common understanding of best techniques.

Third, troubleshooting common issues encountered by the early operator often decides whether we overcome these obstacles and keep growing on this pathway or abandon the technique. It is at this early stage that we are primed to benefit from real-time feedback from proctoring. While availability, payment, and coordination remain challenges for utilizing proctors, perhaps more remote options will become available in the post-COVID world. At a minimum, leaning on our expert colleagues from our community with a phone call or videoconference when we have questions or find ourselves in a bind is a growing part of our interventional culture.

Fourth, reviewing cases after the fact offers the opportunity for growth and improvement, regardless of whether it appeared to go well at the time. While this is typically limited to our own practice environments, #MedTwitter is evidence enough that we want to learn from our peers’ experience both near and far. Hopefully we will make use of other platforms that allow for more comprehensive review and exchange of ideas by our colleagues while we grow in our practice.

## Why change?

The skills of an interventional cardiologist cannot be expected to be mastered in a single year of training but rather continue to grow and evolve in the course of our career and clinical practice. As it relates to percutaneous axillary access, one can exclude this from their clinical practice, but which patients would be left behind? Seto et al discuss relevant points for patient selection, but patient preference is often underestimated. We have had patients refuse transfemoral mechanical support citing bedrest restrictions as so limiting that they would decline the intervention and even choose comfort-guided care as an alternative over preferences on vascular access.

Looking ahead, it’s possible that axillary access carries significant benefit over femoral access or carries currently unforeseen risks, but the data are only as useful as the degree to which our skills are developed. “Alternative” often carries a negative connotation as nonstandard treatment, but as outlined in the document, percutaneous axillary arterial access may be the best choice for some patients. Ultimately, in our efforts to offer patients the best care, having more options at our disposal, provided they are performed safely, is usually the best approach for our patients.

## Declaration of competing interest

The author declared no potential conflicts of interest with respect to the research, authorship, and/or publication of this article.
